# The Way We Do the Things We Do: How Cognitive Contexts Shape the Neural Dynamics of Motor Areas in Humans

**DOI:** 10.3389/fpsyg.2018.01296

**Published:** 2018-07-27

**Authors:** Franck Vidal, Boris Burle, Thierry Hasbroucq

**Affiliations:** Aix-Marseille Université, CNRS, LNC UMR 7291, Marseille, France

**Keywords:** primary motor areas, supplementary motor areas, CNV, preparation, reaction time (RT)

## Abstract

In spontaneously triggered movements the nature of the executed response has a prominent effect on the intensity and the dynamics of motor areas recruitment. Under time pressure, the time course of motor areas recruitment is necessarily shorter than that of spontaneously triggered movements because RTs may be extremely short. Moreover, different classes of RT tasks allow examining the nature and the dynamics of motor areas activation in different cognitive contexts. In the present article, we review experimental results obtained from high temporal resolution methods (mainly, but not exclusively EEG ones), during voluntary movements; these results indicate that the activity of motor areas not only depends on the nature of the executed movement but also on the cognitive context in which these movements have to be executed.

## Introduction

It is widely established that the nature of the recruited motor areas and the time course of their recruitment strongly depend on the nature of the movement to be performed (Krakauer and Ghez, 2000). This is well illustrated in humans by studies using the Bereitschaftspotential (BP). The BP, discovered, by Kornhuber and Deecke (1965), is a slow electroencephalographic (EEG) wave which precedes self-paced spontaneously triggered movements. It is composed of an early, mostly bilateral, component and a late lateralized one. These components are finally followed by a contralateral “motor potential” which develops over the primary motor area (M1) just before electromyogram (EMG) onset [Shibasaki and Hallett (2006) for a review]. For elementary (e.g., brisk finger movement) slow rate self-paced movements, there is a consensus for considering that the surface-recorded premovement components (early BP, late BP, and motor potential) are essentially generated by motor areas (Shibasaki and Hallett, 2006) and the influence on the BP of movement characteristics such as, for example, the nature of the effector (which finger is used, foot, shoulder, hip, knee, tongue, eyes, etc.), the force to be exerted, the speed, the accuracy, or the complexity of the response, etc. have been well documented [Lang (2003) and Shibasaki and Hallett (2006) for reviews; Kukleta et al., 2012].

It is also established that the nature of the movements executed to realize a given action depends on the cognitive context in which this action must be realized. For example, Rosenbaum and Jorgensen (1992) asked subjects to lift a rod in order to transfer it to a final given position. The nature of the performed movement (hand orientation) depended on how comfortable the arm would be at the end of the transport following the lifting movement. Moreover, the choice of a hand grip also depended on the choices that had been used before. Haggard (1998) obtained similar results: the type of movement executed to grasp an object depended on whether this object should only be grasped or of it should be moved afterward.

Now, one can wonder whether, for a given movement, the cognitive context in which this movement is executed has any influence on the nature and the dynamics of the recruitment of motor areas (Mirabella et al., 2008). A key aspect of the cognitive context in which an action develops relates to the nature of the decision leading to the upcoming action. Intentional actions can be classified according to the nature of the decision to be performed before acting: the “… ‘how’ aspects of the act.” (Kukleta et al., 2012, p. 65), its “what,” its “when,” and its “whether” (Brass and Haggard, 2008), or no decision. Therefore, in the following, we will examine the effects, on motor areas [and more specifically on primary motor (M1), dorsal premotor (PMd), and supplementary motor (SMA) areas] in humans of these decisional contexts in which an action develops and will conclude by showing that even more general cognitive states or attitudes regarding the actions to be performed may influence the dynamics of activation of the motor areas.

To access the dynamics of motor areas activation, one must resort to high temporal resolution brain imaging techniques and this is why, although metabolic (PET, fMRI) methods have provided highly valuable information on cortical motor control, we will not discuss much these results here, given their poor temporal resolution.

To give efficient access to most motor structures, MEG, although presenting excellent temporal resolution, presents a serious drawback: it is almost blind to radially oriented generators and, as a consequence, to the surface of the gyri. Once again, this has been clearly illustrated in BP recordings. The early, mostly bilateral, component of the BP is clearly visible on EEG recordings and it is demonstrated, on the basis of intracranial recordings that the supplementary motor area (SMA) is one of its main generators [Ikeda and Shibasaki (2003) and Sochůrková et al. (2006) for a review]. However, the onset of the magnetic counterpart of the BP occurs later and is mainly contralateral (e.g., Nagamine et al., 1996). The authors reasoned that, even if SMA is active in such situation, MEG is probably blind to it. First, if the SMAs are active bilaterally, then, intra-fissural activities generate tangential generators of opposite direction which should mainly cancel each other. Now, the part of the SMAs lying on the mesial part of the convexity, the gyral part of the primary motor areas and most of the premotor areas (PM), correspond to radially oriented generators to which MEG is poorly sensitive. Conversely, EEG is very sensitive to these generators and, as such, the early part of the BP can easily be recorded with this technique. This is certainly why tentative MEG-based generator reconstruction of movement-related potentials by Gerloff et al. (1998) failed to identify a generator in the SMAs and/or bilateral M1 or PM activity on the precentral gyrus (but see Erdler et al., 2000).

For these reasons, among the available high temporal resolution brain imaging techniques, we will mainly (but not exclusively) concentrate on EEG studies in the following. Because of volume conduction (Nuñez, 1981), overlapping effects may deteriorate the spatial resolution of EEG recordings and, secondarily, their temporal resolution (Law et al., 1993; Burle et al., 2015). However, as will be seen in the following, specific solutions may be used when necessary, to overcome this difficulty in the study of motor areas.

## When

### Preparing When to Decide

It is possible to prepare according to the timing of task relevant events. Indeed, Requin et al. (1991) distinguished two, not mutually exclusive, types of preparation: “event” preparation and “time” preparation, time preparation does not correspond to deciding “when” to act but rather corresponds to prepare for “when” to decide. This preparation is classically studied by manipulating the duration of preparatory periods (PPs) of choice reaction time (RT) tasks.

Most PPs of RT paradigms involve a preparatory signal (PS) and an imperative signal (IS). During the delay between the PS and the IS, several preparatory operations take place, among which motor process can occur if a movement may be required.

Absolute accuracy of time estimation decreases proportionally to the increase of the duration to be estimated (Gibbon, 1977). Therefore, short PPs allow a better estimation of the occurrence of the IS than long PPs and, if administered in blocked designs, RTs are shorter after short than after longer PPs (Woodrow, 1914), provided that the PP is longer than 200 ms (otherwise, there is no time enough to get prepared: Bertelson, 1967). Note that this effect holds even when subjects have to make a choice after the IS.

The motor nature of time preparation is not warranted *a priori* since the effects of time preparation on performance have been demonstrated to hold even when subjects do not know in advance which response to execute after the IS. Moreover, Davranche et al. (2011), for example, showed that, in a detection task without any time pressure, time preparation does facilitate stimulus detection.

To examine the possible effect of time preparation on motor structures, Davranche et al. (2007) used transcranial magnetic stimulation to probe cortical and corticospinal excitability during the PP of a between-hand choice RT task: according to the nature of the IS (right or left from a fixation point), delivered at the end of the PP, subjects had to press a button with either the right or the left hand (respectively). They used a short and a long foreperiod. As expected, RTs were shorter after the short than after the long PP. Two indices were examined: the amplitude of the motor potential and the duration of the silent period which follows the motor potential (the silent period is the result of intracortical GABA-ergic inhibition).

The silent period decreased with increasing time until the end of the PP. Given that the silent period corresponds to cortical inhibition, its decrease reveals a release of intracortical inhibition (i.e., a net motor activation). This release of inhibition progressively increased during the PPs and was more pronounced for the short PP. For the short period, the motor potential decreased progressively until the end of the PP, suggesting that, when motor activation is maximal, increased cortico-spinal inhibition secures the development of cortical activation to prevent erroneous premature responding [but see also Duque and Ivry (2009) and Duque et al. (2010) for similar results].

Now, a follow-up study demonstrated the efficiency of this preparation on the reactivity of the motor structures: this preparation selectively speeded up corticospinal motor processes. Tandonnet et al. (2012) examined the time course of M1 excitability after the IS of short and long PPs where no prior knowledge regarding the responding hand was available. They showed that after the IS, the size of the motor evoked potential increased faster after a short than after a long PP, on M1 contralateral to the responding hand; such an effect was absent for ipsilateral M1. Moreover, the difference between contralateral and ipsilateral stimulation occurred earlier for the short PP.

In the same vein, Tandonnet et al. (2003) using Laplacian-transformed EEG data, examined the time course of the motor potential after short and long PPs. They showed that the time separating motor potential onset from EMG onset was shorter after a short than a long PP. This indicated that better time preparation (during short foreperiods) speeds up motor processes at primary motor cortex level.

### Deciding When to Act

Bereitschaftspotential studies have shown that cognitive factors influence the BP and these observations might suggest that the cognitive context influences motor areas. However, this not warranted. Due to volume conduction effects (Nuñez, 1981), the mere analysis of scalp potentials does not allow drawing firm conclusions in this respect, unless it would be clearly established by other methods that the generators of premovement potentials are always confined to motor areas. This is not always the case (Kukleta et al., 2012); for example, spontaneously triggered grasping (Bozzacchi et al., 2012) movements or, more generally, spontaneously triggered movements implying interaction with an object, even when mimicked (Wheaton et al., 2005), generate early parietal pre-movement potentials [Di Russo et al. (2017) for a review].

Fortunately, as indicated earlier, for elementary (e.g., brisk finger movement) slow rate self-paced movements, there is evidence that the generators of the BP are essentially in motor areas (Shibasaki and Hallett, 2006): in this case, it is widely admitted that the early part of the BP begins in the SMAs, and, shortly after, spreads bilaterally into the PMds; finally, the late part of the BP develops in contralateral M1 and PMd.

Using simple spontaneously triggered voluntary movements, it is therefore possible to examine the influence of cognitive contexts (such as timing constraints), to conclude about their influence on these motor areas in internally triggered voluntary actions, as will be the case in the following.

Verleger et al. (2016) asked subjects to produce spontaneously triggered independent simple key-presses. In different experimental blocks of trials, different constraints were imposed regarding the minimum duration separating the upcoming action from the preceding one; no upper limit was set on this duration. Subjects received an error feed-back after a given produced key-press if it occurred too close in time from the preceding one. Verleger et al. (2016) showed that “… BPs did vary in accordance with the temporal constraints [put] on the intervals between movements …”. (Verleger et al., 2016, p. 11).

To explore, may be more directly, the effects of timing constraints on motor structures, Baker et al. (2012) used a temporal reproduction task. Subjects were presented with pairs of tones separated by specific time intervals; afterward, they had to spontaneously reproduce the same interval between two brief button presses at their own choosing. Each button-press was therefore a spontaneously triggered self-paced action. Nevertheless, no timing requirement was imposed to the first action whereas timing control was explicitly required before the second one. It appeared that the BP preceding the second button press was much larger than that preceding the first one, suggesting that attention to elapsed time, “i.e. the process of orienting attention in time towards the moment of movement initiation” (Baker et al., 2012, p. 715), is one of the crucial factors in the elicitation of the BP and, therefore, the dynamics of activation of motor areas. This effect was obvious over the SMAs but absent over contralateral M1. This influence of temporal constraints on the BP, and therefore on motor structures, was consistent with several data sets indicating a prominent involvement of the SMAs in timing [Casini and Vidal (2011) and Coull et al. (2016) for short reviews] and by the fact that the SMA BOLD response correlates with the amount of attention paid to elapsing time (Coull et al., 2004).

**FIGURE 1:**
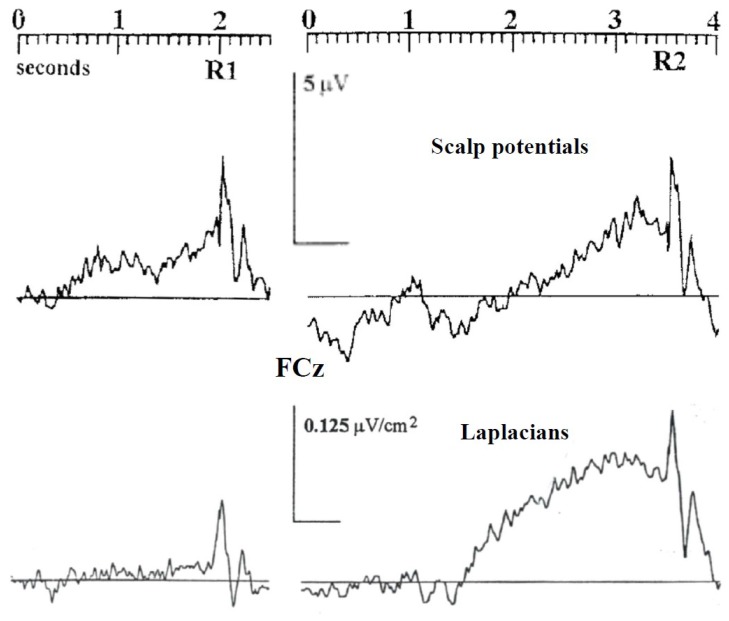
The influence of timing control on the activities recorded over the SMAs. Adapted from Macar et al. (1999). Upper panel: response-locked scalp potentials (reference: right mastoid) recorded over the SMAs before the first (R1) and the second (R2) key presses of a spontaneously triggered interval delimited by two brief button presses (target interval: 2.5 s). Lower panel: same dataset after Laplacian transformation.

Macar et al. (1999) obtained similar results and added supplementary information regarding the sensitivity of motor structures to the timing context. Subjects learned by trials and errors to produce, at the time of their own choosing, a target interval delimited by two brief button presses; they received after each trial a feed-back on their timing performance. As in the Baker et al. (2012) experiment, scalp potentials showed a greater BP after the second action of the sequence than after the first one, especially at midline fronto central electrode (**Figure 1**). Now, to attenuate spatial overlapping effects due to volume conduction (Nuñez, 1981), the authors resorted to the Laplacian-transformation of surface scalp potentials [see Kayser and Tenke (2015) for a tutorial review]. Acting as a high-pass filter, this transformation allows to better separate the activities issued from distinct cortical generators, not only in space but also in time (Law et al., 1993; Burle et al., 2015). Notably, this separation is efficient without any inference regarding the number, the orientation, or any other property of these underlying generators. Surface Laplacian revealed to be efficient in separating activities generated by sensorimotor areas from those generated by frontomesial structures, including the SMAs.

Once Laplacian-transformed, EEG data revealed qualitatively different patterns of activation before the first action and the second one. Whereas a very large BP was evidenced over the SMAs before the second action, no measurable activity was elicited before the first one (**Figure 1**). On the contrary, large BPs were evoked over contralateral and ipsilateral M1s before the first and before the second action. This indicates that, in the Macar et al.’s (1999) experiment, and probably in Baker et al.’s (2012) one, the amplitude differences observed at the midline frontocentral sites on scalp potentials between the first and the second action can be explained by a strong participation of the SMAs to the BP before the second action and no contribution of the SMAs to the BP before the first action. The activity observed over the SMAs before the first action on scalp potentials was very likely volume conducted from bilateral M1 and/or PMd generators given that M1s and/or PMds were involved both before the first and the second action. One might argue that the Laplacian transformation does not solve the inverse problem, which is true. However, whatever the exact contribution of, say, the cingulate cortex areas, to the activities recorded right over the SMAs, the “flat” trace observed before the first action in Macar et al.’s (1999) data, indicate that at least the SMAs did not contribute to the BP and, therefore, to the activity of the motor areas, when triggering the first self-paced action of the sequence.

Of course, in this kind of situation, as stressed by Baker et al. (2012) or Macar et al. (1999), the status of the first press differs from that of the second press in that no specific timing constraint is required before the first action, whereas once this action has been triggered, the occurrence of the second one must be finely timed. It can therefore be concluded that the cognitive context regarding “when” to act has a strong influence on the dynamics of motor areas and, more specifically, of the SMAs.

## What

Although there have been clever attempts to test the influence of the “what” (e.g., Dirnberger et al., 2000) and “whether” (Brass and Haggard, 2007) components of intentional action, on the dynamics of motor structures using BP recordings, the contribution of non-motor structures to pre-movement activities (e.g., prefrontal areas:) is not unlikely (Dirnberger et al., 2000; Brass and Haggard, 2007). Moreover, there are more convenient situations to explore the “what” and the “whether,” which have been used for more than one century. These correspond to RT situations.

This is why we will mainly concentrate on RT tasks, in the following, for evaluating the effects, on motor structures, of the cognitive context related to the “what” (and later on the “whether”) component of response decision. In the following, we will argue that even for very simple voluntary movements such as, for instance, pressing a button, the dynamics of motor areas can depend on the cognitive context in which these very simple movements have to be executed after the response signal (RS). Contrary to voluntary internally triggered movements, such movements are externally triggered by the RS according to an arbitrary stimulus-to-response mapping rule (given by instructions). It is to be noted that, in general, these simple movements, although externally triggered, are not externally driven as would be the case for, say, tracking a moving target or seizing an object on a table, which control may reveal to be more automatic (bottom-up), once the subject is appropriately trained (Scott, 2016).

### Preparing What to Decide

A very popular paradigm to manipulate subjects’ knowledge regarding “what” has to be done is the precueing paradigm (Rosenbaum, 1983) and its variants. In this paradigm, the PS can deliver complete, partial, or no information regarding the characteristics of the movement (if any) required by the IS. More abstract information can also be delivered by the PS (for example, about the nature of the stimulus-to-response association). In these conditions, scalp EEG slow negative shifts are evoked during the preparatory interval. The ensemble of these shifts has been named contingent negative variation or CNV (Walter et al., 1964). Precues have a clear influence on the amplitude of the CNV. However, it has been recognized, very early, that the CNV is a complex activity involving several classes of generators, including motor and non-motor structures which, of course, because of volume conduction, often mix at scalp level. To overcome this difficulty the use of the lateralized readiness potential (LRP) has been proposed (e.g., Gratton et al., 1988). If a right-hand or a left-hand response are possible, the CNV may become larger over the hemisphere contralateral to the responding hand when prior knowledge (precue) regarding the responding hand is delivered by the PS (the lateralization occurs after the IS if no precue regarding the responding hand is available) (Kutas and Donchin, 1980). Therefore, the lateralized part of the CNV, during the PP, or the lateralized part of post-IS ERPs (called the LRP) have been assumed to correspond to motor channels activation, generated in contralateral M1 or at least contralateral motor structures (as it is the case for the lateralized part or the BP before elementary movements). Based on this reasoning, it has been concluded that examining the effects of information conveyed by precues on the LRP should allow examining precueing effects specifically on motor structures. However, although asymmetric activation of motor structures do generate LRPs, it has been shown that lateralized readiness activities during PPs may also be generated by non-motor processes generated by non-motor cortices (e.g., Verleger et al., 2000; Praamstra et al., 2005; Mathews et al., 2006; Praamstra and Kourtis, 2010). Therefore, because of volume conduction, motor and non-motor activities may mix at scalp level and may render precueing effects on the LRP difficult to interpret in terms of activation of the motor structures^1^.

It is to be noted, as shortly indicated in the section “Introduction,” that this “mixing” problem is not specific to LRP studies. Indeed, although EEG is a very suitable technique to study the time course of brain activities, volume conduction may result in overlapping effects in space which may cause, secondarily, a deterioration of EEG temporal resolution (Law et al., 1993; Burle et al., 2015); therefore, specific solutions must be implemented in EEG studies to cope, when necessary, with this difficulty. Sometimes it may be necessary to resort to intracranial recordings. For example, electrocorticography has proven efficient to clearly separate the activities from M1 and PMd in humans (Mattia et al., 2012) while intracerebral recordings (local field potentials) have proven efficient to clearly separate the activities of SMA proper from those of pre-SMA (Bonini et al., 2014). Fortunately, as already indicated, it is not always necessary to resort to intracranial recording in humans to improve the spatial and, as a consequence, the temporal resolution of electrophysiological recordings in humans: it is possible to separate quite efficiently the activities issued from left, right, or medial motor structures using the Laplacian transformation [e.g., Vidal et al. (2003) for an illustration], and, when using a realistic model of each subject’s head, it may even provide an “… estimate of the electrical potentials that would be recorded near the cortical surface” (Gevins et al., 1995). In the following, all the reported EEG-based precueing experiments used the Laplacian transformation to examine the effect of different precues on motor (and non-motor) areas. In all cases, precues modified RTs, as could be expected from the relevant literature.

#### Force and Direction

MacKay and Bonnet (1990) examined the effect of prior information (“what”) regarding the force (weak or strong) and the direction (flexion or extension) of an elbow movement, during a 1.5 s PP. The authors showed that prior knowledge about force or direction increased sensorimotor areas activities during the PP, but not the activities of mediofrontal motor areas, including the SMAs (recorded at central midline site). The effect of directional precue on M1 activity was predicted based on a previous stretch-reflex experiment conducted by Bonnet (1983), as will be developed now.

Although the short-latency stretch reflex has a purely spinal origin, long latency stretch responses (LLSR) are, now, admitted to involve oligosynaptic transcortical long loop responses which efferent pathway originates in the motor cortices [Matthews (1991) for a review]. Bonnet (1983) studied the effect of prior knowledge regarding the direction of a wrist movement, on the amplitude of the stretch reflex. Subjects had to perform either a wrist flexion or a wrist extension with their right hand. In some trials, a stretch was applied to the flexor muscles and this stretch could be applied at different moments of the 1 s PP. When the PS indicated in advance that the movement required by the IS would be a flexion, the late component of the LLSR of the flexor muscles increased; when the PS indicated an extension, the same late component decreased. Moreover, these differential effects progressively increased with time during the PP and were maximal just before the IS. Given that the efferent pathway of the LLSR originates in the motor cortices, advance information regarding the nature of the muscles involved in the response generated a progressive increase of excitability of the cortical neurons controlling the acting muscles, or a progressive decrease of excitability of the cortical neurons controlling the muscles antagonists to the required response; this activation/inhibition pattern of the required/non-required responses was reproduced later (Bonnet et al., 1991). Of course, this activation/inhibition pattern might represent a special case, due to the mutually exclusive nature of the activation of antagonist muscles in flexion/extension movements (see below for discussion). Nevertheless, it remains that the effect of prior knowledge regarding the nature (flexion/extension) of the upcoming movement has a strong influence on M1s activity, and this influence is functionally relevant to the task since it was found to be larger in fast (short RT) performers than in slow performers (Bonnet, 1983).

#### Duration of a Short Motor Sequence

Vidal et al. (1995) studied the time course of preparatory activities over contralateral primary motor areas, contralateral parietal areas, and mediofrontal areas including the SMAs, as a function of prior knowledge regarding the duration (short: 700 ms or long 2500 ms) of an interval delimited by two brief button presses. In other word, the choice was between a short or a long motor sequence. In the early part of the PP, the precues evoked no effect over contralateral M1s, whereas this same precues had a strong and sustained influence over the SMAs. The reverse occurred at the end of the PP: as time elapsed during the PP, the sensitivity of mediofrontal activities vanished. In the same time, activities recorded over contralateral M1s became sensitive to the precues. This exchange in activation across areas suggests that advance preparation began in the mediofrontal motor areas including the SMAs and that information regarding this preparation was transferred to contralateral M1 in the last phase of the PP.

It is interesting to note that over the contralateral parietal areas, contrary the effects observed over motor areas, the precues did not evoked any effect at any time during the 2 s PP.

#### Relative and Overall Durations of a Short Motor Sequence

**FIGURE 2:**
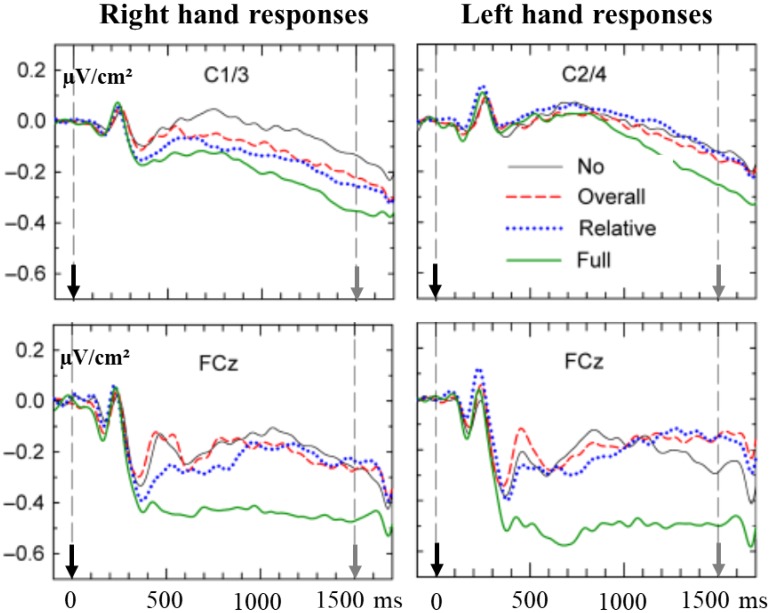
Preparatory effects recorded over the SMAs (lower panel) and M1s (upper panel) before a right hand (left panel), or a left hand (right panel) response, depending on the nature of a precue: No prior information solid black line, information about “relative duration” dotted blue line, information about “overall duration” dashed red line, and full information solid green line (see text for more details). Adapted from Leuthold and Jentzsch (2011). - The upper left panel corresponds to recordings over left M1 (C1/C3) before a right-hand response. - The upper right panel corresponds to recordings over right M1 (C2/C4) before a left-hand response. - The lower left and right panels correspond to recordings over the SMAs (FCz) before a right- or a left-hand response, respectively. On each panel, the black arrow indicates the occurrence of the PS (0 of time) while the gray arrow indicates the occurrence of the response signal (at 1500 ms).

Leuthold and Jentzsch (2011) extended these results. In a RT task, subjects had to produce one among four possible sequences of two consecutive key presses. The produced sequence could be either an overall short (500 ms) or an overall long (800 ms) sequence and in each sequence, the duration of the first key press either had to correspond to one-third of the second one or had to last three time more than the second one. The IS was preceded by a PS which could either deliver advance information on overall duration (500 or 800 ms), on relative duration (1:3 or 3:1) or both. The PS could also provide no prior information. The right or the left hand were used in different blocks of trials. A very large activation was observed from the beginning of the PP over the SMA (**Figure 2**) when full advance information was available (overall duration and ratio between first and second press). The comparison between early mediofrontal and M1s activation was not of interest for the purpose of the study and, therefore, was not explicitly reported by the authors; however, from **Figure 2**, it seems that medial sensitivity to the full precue developed very early over the SMAs while it seemed to develop later over the left M1 (whatever the responding hand). Moreover, the activity recorded over the left motor areas was sensitive to partial advance information (overall duration or ratio). Finally, it is interesting to note, as stressed by the authors, that contralateral lateralization was evidenced only after full information. This suggests that prior knowledge regarding the “what” may or may not be conveyed to motor structures, depending on the availability of other complementary information. This suggests the existence of a hierarchy in the motor implementation of prior information regarding the properties of upcoming responses, i.e., motor areas cannot get prepared to certain response characteristics unless prior knowledge regarding other ones is available.

#### Complexity, in Temporal Order, of a Short Motor Sequence

Leuthold and Schröter (2011) studied the effect of prior information regarding motor sequences that differed in structural complexity but not in length or number (and nature) of involved effectors. When hand and sequence were precued, additional prior knowledge about the structural complexity of the upcoming sequence unequivocally influenced mediofrontal motor structures but leaved M1s insensitive. M1 sensitivity to structural complexity could be evidenced later, after the IS, just before response execution. This sequential sensitivity, between mediofrontal areas and M1s, to sequence complexity, for temporal order, is somewhat similar to the sequential sensitivity to response duration, between mediofrontal areas upstream and primary motor areas downstream (Vidal et al., 1995). These sets of results also suggest that, for temporal order and response duration, there is a functional hierarchy in the implementation of these response characteristics between mediofrontal motor areas, upstream and primary motor areas, downstream.

A comment is in order here. From what precedes (regarding the effects of prior information on motor areas) it is an empirical fact that advance (partial or complete) information regarding specific features of incoming movements does influence the dynamics of motor areas when they get prepared for responding; however, this does not necessary means that these features are coded as such by motor structures. In other words, this does not mean that response parameters or other types of movement characteristics (such as for example the detailed pattern of muscle contractions required to achieve the movement) are represented at the level of motor areas. Certain experimental results are compatible with the view that response parameters and/or kinetic movement properties are coded by motor cortices (e.g., Riehle and Requin, 1989; Kalaska, 2009; Milekovic et al., 2015), while other ones (e.g., Shenoy et al., 2013; Kao et al., 2015) suggest that the evolution of neural activity “… should be best captured not in terms of movement parameter evolution, but in terms of the dynamical rules by which the current state causes the next state” (Shenoy et al., 2013, p. 340).

Given that the brain has probably more than one string to its bow, it is not impossible that the response to this important matter may depend on the nature of the control exerted to trigger and/or execute the ongoing movement: bottom up or top down [see Scott (2016) for a clear distinction between these two classes of motor control]. Now, whatever the issue of this controversy (which, nevertheless, is out of the scope of the present article), the sensitivity of motor areas to prior information regarding specific response features allows concluding that the dynamics of these areas is influenced by prior knowledge, that is, by the cognitive context in which these movements are to be executed. Moreover, the fact that motor areas cannot get prepared to certain response characteristics unless prior knowledge regarding other ones is available (Leuthold and Jentzsch, 2011) indicates that motor areas cannot be influenced by just any information regarding upcoming actions. This might put certain constraints regarding the way movements are controlled by motor structures.

### Deciding What to Do

During the short time scale (about 500 ms or less) of RT, post-stimulus and pre-response ERPs tend to overlap, due to the joint effects of (1) their temporal proximity and (2) volume conduction (Kutas and Donchin, 1980). As mentioned earlier, to isolate motor components from this ERPs mixture, the use of the LRP has been proposed. We have already presented some difficulties encountered with the interpretation of this measure as a “purely motor” index. Besides these difficulties, another problem has been identified since the very beginning by Gratton et al. (1988, p. 339): the LRP cannot “... distinguish cases in which one response is activated from cases in which the other response is inhibited …”. What was a theoretical statement at that time turned out to be empirically verified in RT conditions.

**FIGURE 3:**
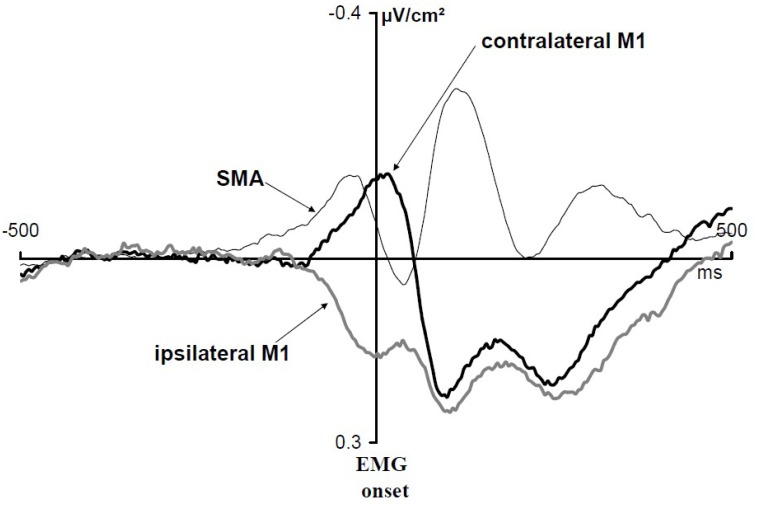
EMG-locked ERPs recorded over M1s and SMAs in a between-hand choice RT task. Adapted from Vidal et al. (2003). Activation of contralateral M1s (black thick line), inhibition of ipsilateral M1s (gray thick line), and N-40 (black thin line). Laplacian-transformed data.

Vidal et al. (2003) asked subjects to perform a between-hand choice RT task. They studied Laplacian-transformed ERPs activities over motor areas. These ERPs were time locked to EMG onset (**Figure 3**). Over M1s contralateral to the responding hand, a transient negativity developed before and culminated shortly after EMG onset. Over M1s ipsilateral to the responding hand a transient positive deflection began shortly before and lasted until shortly after EMG onset [but see also Amengual et al. (2014) or van de Laar et al. (2012)]. Several pieces of evidence indicate that the negativity corresponds to contralateral M1 activation while the positivity corresponds to ipsilateral M1 inhibition [see Burle et al. (2004) for a detailed discussion]. Before this activation/inhibition pattern, another component could be evidenced over the SMAs (**Figure 3**). This component began, peaked and resolved before the beginning, the peak, and the resolution of M1 activation, respectively. It was called N-40 because it culminates about 40 ms before EMG onset [but see also Mansfield et al. (2012) who discovered independently this same component and named it N-120 by reference to response-locked data instead of EMG locked]. Therefore, when a decision regarding what to do must be taken under time pressure, both M1s are involved (although very differently) as well as the mediofrontal motor structures (recording electrodes placed over the premotor cortices did not evidence any specific components before EMG onset). Note that these components are perfectly mixed on scalp potentials recordings and cannot be identified unless separation methods (here Laplacian transformation) are applied [see Burle et al. (2015, figure 7) for a demonstration].

Tentative source localization identified the generator of the N-40 within the SMAs (Carbonnell et al., 2013). Inverse problem solutions must always be interpreted cautiously; however, these localizations used two independent inversion methods, both pointing to a quite superficial generator, consistent with the fact that Laplacian-transformed data hardly pick up deep sources activities. Therefore, it seems rather safe to admit that the generator or, at least, the main generator of the N-40 is a superficial one lying in the SMAs. Although the Laplacian transformation dramatically improves the spatial (and temporal) separation of distinct generators, this improvement is not sufficient, however, to distinguish between the subdivisions of the SMAs, namely pre-SMA and SMA-proper (Luppino et al., 1991; Matsuzaka et al., 1992) and the inversion methods did not allow either to separate the activities of these two subdivisions. However, Ramdani et al. (2018) showed that the amplitude of the N-40 was reduced by acute dopamine depletion, provoked by diet-induced depletion in the precursors of dopamine synthesis (McTavish et al., 1999), namely tyrosine and phenyl-alanine. On the other hand, neither Larson et al. (2015) nor Ramdani et al. (2018) found any reduction of the error negativity (Falkenstein et al., 1991; Gehring et al., 1993), a response-related ERP for which amplitude is modulated by performance (it is usually large on errors and small on correct trials; Vidal et al., 2000). Now, being established by intracerebral recordings in humans that the generator of the error negativity or at least its main generator, lies in the SMA proper but not in the pre-SMA (Bonini et al., 2014), one can conclude that SMA proper activity is not noticeably impaired by acute dopamine depletion. Therefore, the dissociation between the N-40 and the error negativity as regards their sensitivity to dopamine depletion, suggests that the generator of the N-40 (or at least its main generator) lies in pre-SMA but not in SMA-proper.

Two main differences show up between spontaneously triggered movements or preparatory process, on the one hand, and externally triggered movements to be chosen under time pressure, on the other hand. The first one is obvious: even though there is an additional decision to be taken regarding “what” in choice conditions, the time course of motor areas activations develops in much shorter time ranges: no more than 300–400 ms before EMG onset. The second one is motor inhibition. To our knowledge, in spontaneously triggered movements, no inhibition of the non-involved primary motor cortex has been described. For example, in the Dirnberger et al.’s (2000) study, participants, on each trial, had either to alternate or to spontaneously choose which hand should spontaneously be moved. No sign of inhibition at M1 level was evidenced, although the extra activation recorded over the SMA in the choice condition was interpreted by the authors as a need for inhibition.

Of course, during a PP, Bonnet (1983) evidenced an activation/inhibition pattern between flexors and extensors at M1 level, via transcortical long-loop reflexes. However, this may have resulted from some sort of reciprocal inhibition between agonistic and antagonistic muscle commands, which are mutually exclusive, if flexion or extension is required. In the present case, each hand is not “naturally” antagonistic of the other one. Instructions only, render them mutually exclusive.

We will see now that ipsilateral M1 inhibition is not the mere by-product of a variant of (hard-wired) reciprocal inhibition between right and left M1s, but that it is driven by the cognitive context of the task.

In some situations, the decision process must also take into account not simply “what” to do but also the likelihood of each possible response. Meckler et al. (2010) compared a standard between-hand choice RT task to a “biased” one. In the biased task, one of the two possible responses was frequent (80%) and the other one was rare (20%). In cognitive terms, one response was expected, while the other one was unexpected (expectancy was manipulated between-blocks). In the standard task (50% right 50% left) no specific expectation could be drawn from the situation.

As could be anticipated, no effect of expectancy was observed over contralateral M1. Indeed, there is good evidence from corticograms recorded in monkeys as well as in humans that the motor potential (contralateral negativity) represents the activation of M1 contralateral to the responding hand [see Vidal et al. (2003) or Burle et al. (2004) for a discussion on this point].

On the contrary, response expectancy had a clear effect on ipsilateral inhibition, i.e., on inhibition of M1 involved in the not to be given response. It was very small (yet present) when subjects produced the expected response (hence inhibited the unexpected one), very large when subjects produced the unexpected response (hence inhibited the expected one), and just in between when no specific expectation could be drawn (i.e., standard choice condition). We concluded that ipsilateral inhibition is a context-dependent component representing a pro-active control of errors, that is, a mechanism aimed at preventing the risk of committing an error. Consistent with this interpretation, was the fact that, in the unexpected condition (where ipsilateral inhibition was the strongest), there was a negative correlation between the size of this component and the error rate: subjects who presented the strongest inhibitions were those who presented the smaller error rates.

Back to the problem identified by Gratton et al. (1988) regarding the use of the LRP and the possible confound between contralateral activation and ipsilateral inhibition, the results of Meckler et al. (2010) demonstrate that this theoretical concern turned into a concrete difficulty.

**FIGURE 4:**
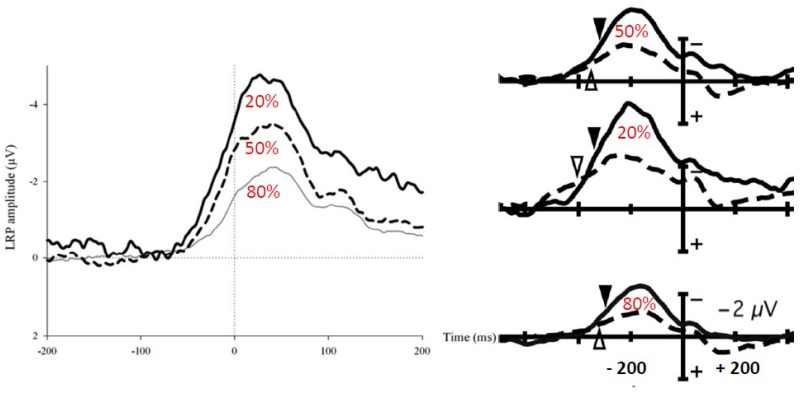
Effects of a probability bias on EMG locked LRPs. The LRP corresponds to the difference between activities recorded over M1 contralateral an ipsilateral to the response. Left panel [adapted from Vidal et al. (2015)]: EMG-locked LRPs computed from the data reported in Meckler et al. (2010) in a between-hand choice RT task. Each trace corresponds to expected responses (80% thin line), no-expectation responses (50% dashed line), and unexpected responses (20% bold line). Right panel [adapted from Luck et al. (2009)]: response-locked LRPs from Luck et al. (2009) in healthy controls (solid lines) and schizophrenic patients (dashed lines). Lower traces correspond to expected responses (80%), middle traces correspond to unexpected response (20%), and upper traces correspond to no-expectation responses (50%). The latency differences between left and right panel are due to EMG locking and response locking on the left and right panels, respectively.

Luck et al. (2009), also compared the effects of expectancy on motor ERPs (also with 50/50 and 80/20 probabilities in different blocks of trials) but they did so using the LRP in healthy control subjects and schizophrenic patients. **Figure 4** (right panel) shows that the LRP increases from expected to unexpected responses, the no expectation condition being in between. The authors interpreted their results in terms of motor activation “… when a given stimulus category is expected, the appropriate response can be prepared before stimulus onset and less stimulus-triggered response activation (and hence less LRP) may be needed once the stimulus has been presented …” (p. 7). Scalp potential LRP-transformed data from Meckler et al. (2010) were calculated by Vidal et al. (2015), and it appears from **Figure 4** (left panel) that they obtained exactly the same time course and patterns of activity as those reported by Luck et al. (2009). Therefore, contrary to the interpretation given by Luck et al. (2009), the LRP decrease with response expectancy was not due to decreased activation of the required response. On the contrary, this decrease was due to decreased inhibition of the non-required response.

Schizophrenic patients in Luck et al. (2009) showed decreased LRPs when compared with healthy subjects in all conditions (**Figure 4**, right panel). It is not possible to draw any functional or physiological interpretation from this observation because this effect could be due to decreased activation, decreased inhibition, or both. “Simple” effects of conditions on the LRP correspond, in fact, to interactions between sites (contralateral/ipsilateral) and experimental conditions. Therefore, no firm conclusions can be drawn regarding the causes of these interactions.

**FIGURE 5:**
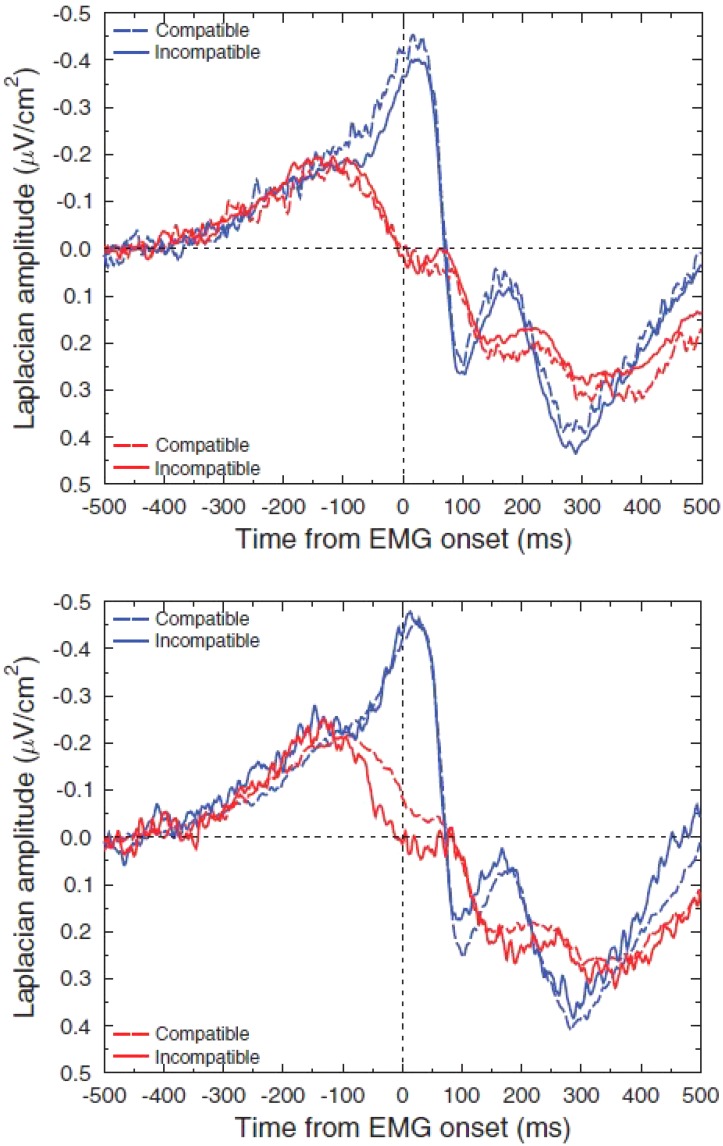
Effect of the probability of compatible and incompatible trials on ipsilateral inhibition. Adapted from Burle et al. (2016). Upper panel: activation (M1 contralateral to the responding hand: blue lines)/inhibition (M1 ipsilateral to the responding hand: red lines) pattern in compatible (dashed lines) and incompatible (solid lines) trials when incompatible trials are highly probable. Lower panel: activation (M1 contralateral to the responding hand: blue lines)/inhibition (M1 ipsilateral to the responding hand: red lines) pattern in compatible (dashed lines) and incompatible (solid lines) trials when compatible trials are highly probable.

Manipulating the probability of a response modulates incorrect response inhibition. A question remains as to when this modulation is set. Indeed, in the studies quoted above, the participants knew at the beginning of each block the probability bias. They could have hence set *a priori* an asymmetric inhibition coefficient, explaining the observed modulation. Burle et al. (2016) evaluated whether this modulation is set *a priori* or is modulated online as a function of the context. Instead of manipulating the probability of individual responses, they manipulated the probability of compatible and incompatible trials (30% vs. 70%), in a Simon task [Simon (1990) for a review]. In the rare-incompatible condition, participants tend to activate the response ipsilateral to the stimulus. When an incompatible trial occurs, the risk of an error is hence very high. On such trials, inhibition was larger on incompatible than in compatible trials (**Figure 5**). On the contrary, in the frequent-incompatible condition, in which the risk of committing an error was low, no effect of compatibility was observed on ipsilateral inhibition.

It must be stressed that in this experiment, all responses (right and left) and stimulus positions were equiprobable. Hence, the level of required inhibition had necessarily to be set after the IS, since compatibility could vary from one trial to another. In other words, high expectancy regarding compatibility did not result in a constant ipsilateral stronger inhibition for all the trials but in fast stimulus-triggered trial-by-trial adjustments of inhibition to congruency. This demonstrates the high versatility of these knowledge-dependent (i.e., cognitive-pendent) motor effects.

## Deciding: “How,” “Whether,” or Not Deciding at All

### How

In several RT task situations, the stimulus-to-response (S–R) mapping is arbitrary and necessitates applying a rule. This rule specifies *how* to transform a given stimulus into an appropriate response. The mapping rule can be quite easy or difficult to apply, easiness being judged by its effect on RTs and error rates. This is on this aspect of the “how” (to transform a given stimulus into an appropriate response) that will focus now.

Carbonnell et al. (2002) asked subjects to press a right or a left button according to the IS: a word centrally presented on a screen. The words could be either “droite” or “gauche” (right or left in French). Two seconds earlier, the PS could either be the same word as the IS or the word “neutre” (neutral in French). This last PS conveyed no prior information regarding the responding hand. Two different groups of subjects had to apply two different S–R mapping rules. The easy rule consisted in pressing the right button in response to the word “right” and the left button to the word “left.” This condition was said “compatible.” The uneasy rule was opposite: right response to the word “left” and left response to the word “right.” This condition was said incompatible.

When no precue was available (PS: “neutral”) RTs were longer for the incompatible group than for the compatible group. When a precue was available no RT differences were observed between the two groups. This latter result is not unexpected since it is assumed that when precued, the S–R mapping (or response selection operation) is completed during the PP.

**FIGURE 6:**
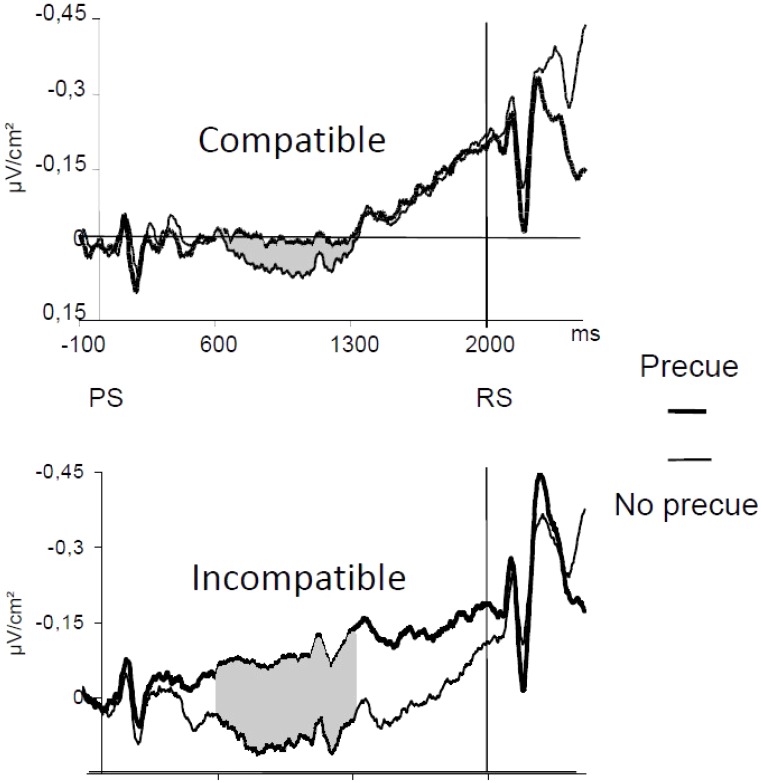
Effect of stimulus response compatibility on the sensitivity of the CNV to advance information. Adapted from Carbonnell et al. (2002). CNVs recorded over the SMAs (FCz) as a function of precueing: bold lines: hand precued, thin lines hand uncued. Upper panel: compatible mapping. Lower panel: incompatible mapping. PS, preparatory signal; RS, response signal.

When the responding hand was precued, no effect of the precue could be evidenced above the primary motor areas during the PP, neither in the compatible, nor in the incompatible group. A different picture showed up over the mediofrontal motor areas. Activity was slightly (yet non-significantly) larger in the precue than in the no-precue condition for the compatible group. On the contrary, at the same recording site, a very strong and long-lasting effect of the precue was observed for the incompatible group (**Figure 6**). Therefore, the nature of the transformation of a given stimulus into a given response has a strong influence on mediofrontal areas (including the SMAs), but let primary motor areas insensitive, suggesting that SMAs are specifically involved in response selection, acting upstream to M1s, which would be more concerned by execution processes. One might argue that not only do response selection processes can take place during the PP, but also response programming, which is true. However, in the present case, the movements to be performed were identical; as such, the programming operations needed to prepare them was not different between groups. Therefore, the interaction observed between group (compatible/incompatible) and precueing (precued/uncued) factors could be attributed to nothing but the nature of the S–R mapping rule to be applied, i.e., solving the “how” problem.

### Whether

The prototypical paradigm in which the “whether” component of response decision is best explored in RT tasks is the Go Nogo task. In these tasks, only one response is possible and, according the nature of the IS, subjects have to choose whether or not they will respond. At variance with Brass and Haggard (2008) assumption, it has been proposed that deciding between executing a response or withholding it involves the same (Nieuwenhuis et al., 2003; Gomez et al., 2007) or, at least similar (Verleger et al., 2006) types of motor decision as deciding to execute one among several responses.

**FIGURE 7:**
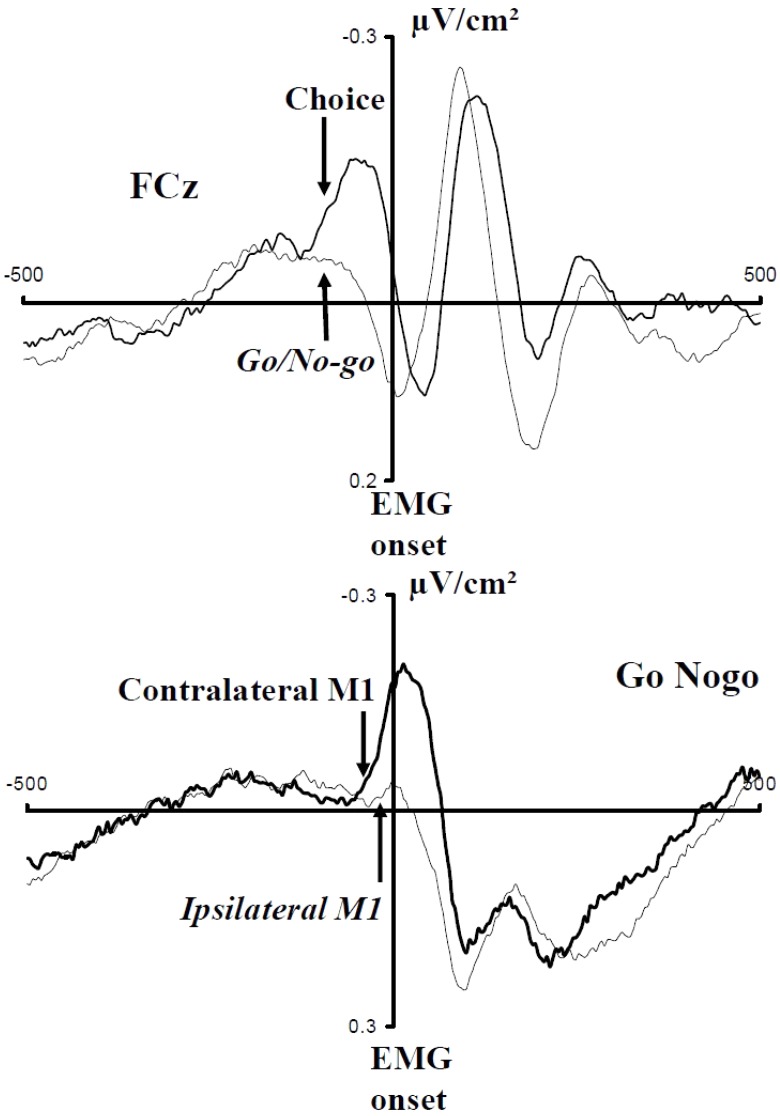
Effect of the nature of the decision to be performed (Choice vs. Go-Nogo). Adapted from Vidal et al. (2011). Upper panel: activities recorded over the SMAs in Choice (bold line) and Go Nogo (thin line) tasks; note the absence of a N-40 in the Go Nogo task and its presence in the choice task. Lower panel: activities recorded over contralateral (bold lines) and ipsilateral (thin lines) M1 in a Go Nogo task; note the absence of inhibition before EMG onset.

Vidal et al. (2011) examined the pattern of activation of SMAs and M1s in a between-hand choice RT task and a Go Nogo task, performed in different blocks of trials. Their results are in line with Brass and Haggard (2008) assumption. Deciding “what” or deciding “whether” involve qualitatively different processes. Considering that there was no risk of committing an error, no ipsilateral inhibition was expected and, indeed, there was no sign of ipsilateral inhibition in the Go Nogo task, contrary to the choice condition. More important, contrary to the choice task, there was no N-40 in the Go Nogo condition (**Figure 7**). This shows that the SMAs were not involved in the decision required in the Go Nogo task and suggests that the N-40 is a physiological sign of choice (and/or motor programming) between alternative movements.

But what about activation? In the Go Nogo task, activation was significantly shorter (more phasic) than in the choice condition and, indeed, the time separating EMG onset from the mechanical response was longer in the choice than in the Go Nogo task. Therefore, in the comparison between “whether” and “what,” the build-up of the motor command is also affected, and this results in different execution times. This is in line with observations reported by Ulrich et al. (1999) who showed that Go Nogo tasks yield more forceful responses than choice RT tasks.

In other words, even the motor command can be affected by the decisional context in which this command must be issued and executed.

Another interesting situation can be informative about the “whether” element of response decision: the Stop Signal paradigm (e.g., Logan and Cowan, 1984). While in the Go Nogo tasks the IS can require to withhold a prepared response, in the Stop task, the IS always indicate a response. However, in a certain proportion of trials, the IS is followed by another signal (the so-called “stop” signal) requiring withholding the response indicated by the IS. RTs of correctly executed responses have been shown to be influenced by the possible presence of these stop signals: RTs are longer on go trials of a stop task compared to (classical) go-only tasks (e.g., Mirabella et al., 2006). However, this contextual increase in RT does not necessarily mean that motor processes or motor structures are concerned. Mirabella et al. (2008) went a step further and examined the execution of hand-reaching movements in a stop and a non-stop context in humans. They observed again that RTs of correct go responses of a stop task were longer than RTs of a go-only task. More important, they evidenced an increase in the movement time, i.e., the time separating movement onset from target reaching. It is very likely that movement time corresponds to the involvement of motor structure during execution, and/or completing “… of the motor plan during the execution of the reach” (Mirabella et al., 2008, p. 1007). It is to be noted that contrary to Go Nogo tasks where only one go response is possible, a choice is required in the go trials of a stop task, as it is the case in a (classical) go-only task. Therefore, the differences in RT and/or movement time evidenced by Mirabella et al. (2008) cannot be attributed to a difference in the number of possible responses. As a consequence, these results indirectly show that knowledge about the possible occurrence of a stop signal after the IS (i.e., cognitive context) has a strong influence on (at least) motor areas functions on go trials (Mirabella et al., 2008).

Besides motor areas, subcortical structures certainly take part to contextual effect and, interestingly, it has been shown that the subthalamic nucleus is causally involved in stop-related context-dependent effect (Mirabella et al., 2013); however, this is clearly out of the scope of the present review and we will not develop further the implication of motor-competent subcortical structures in contextual effects.

### Not Deciding at All

In certain conditions there is no decision at all to be taken during the RT period, regarding the upcoming response. These situations regarding decisional processes can be found in simple RT tasks. Burle et al. (2004) presented data obtained in a simple RT task (figure 7 on p. 159 of Burle et al., 2004). As can be expected from what precedes, no inhibition was evidenced over ipsilateral M1 and no N-40 was evidenced over the SMAs.

Now, Carbonnell et al. (2004) used a precueing paradigm in which the PS either precued which response (right or left) should be executed after the IS or did not precue the responding hand. Precue and no-precue trials were presented in pseudo random order. Therefore, at the occurrence of the IS, subjects had to decide which response should be executed in the no-precue condition while in the precue condition, no choice was necessary if we admit that decision took place during the 2 s PP. In other words, one may consider that precue trials functionally correspond to a simple RT task while no-precue trials functionally correspond to a choice RT task.

According to these assumptions, one should expect ipsilateral inhibition preceded by a N-40 in the no-precue condition and neither ipsilateral inhibition nor N-40 in the precue condition. The no precue-condition conforms to these predictions but this is not the case for the precue condition. In the precue condition, a very small but significant inhibition developed over ipsilateral M1 and a small, short-lasting but significant, N-40 also developed over the SMAs. Of course, there was a very large difference between these inhibitions and N-40s, between the precue and no precue conditions. Nevertheless, contrary to what happened in the blocked simple RT task or in the blocked Go Nogo task, N-40 and ipsilateral inhibition had not completely vanished. We reasoned that this could, at least in part, be due to the fact that precue and no-precue trials were mixed in the same blocks of trials. It is possible that in the precue condition, even though no choice was necessary after the IS, the contralateral response still belonged to the repertoire of the possible responses and, as such, required a small amount of, “by security” decision and inhibition. In other words, in the precue trials there would persist a kind of “cognitive remanence” of the S–R mapping requirements to be applied in no-precue trials.

But what about activation? There was no reliable difference between choice (no precue) and simple (precue) conditions over M1 contralateral to the response. Given what has been obtained in the Choice vs. Go Nogo comparison, this may appear surprising. Nevertheless, this, again is in line with the observations reported by Ulrich et al. (1999), who showed that although Go Nogo tasks yield more forceful responses than choice tasks, the force developed in choice tasks does not differ from the force developed in the simple tasks. This later point is consistent with the fact that the EMG bursts (from which the exerted force results) were identical in precue and no-precue conditions in Carbonnell et al.’s (2004) experiment.

The antecedence of sensitivity of SMAs to certain precues during PPs, compared to M1s, the antecedence of SMAs and PMds activation in spontaneously triggered movements or in RT tasks, compared to M1s and finally the disappearance of SMAs activation when no choice is required in RT tasks, suggest the existence of a hierarchy between motor areas, M1 being downstream. But this question is far from being definitely settled, and this view has been strongly challenged [see for example, Cisek and Kalaska (2010) or Mirabella (2014) for a distributed conception of motor decisions].

Moreover, although this article focuses on the sensitivity of motor areas (M1, SMA, and PMd) to cognitive context, we do not mean in any way that these are the only structures to be influenced by this context or that they are the only structures concerned with the decisional context (as indicated before): for example, parietal (e.g., Wheaton et al., 2005), or prefrontal areas (e.g., Brass and Haggard, 2008) may be concerned, as well as subcortical structures (e.g., Mirabella et al., 2013).

## More General Cognitive Attitude *Vis-À-Vis* the Upcoming Action

### Motivation

In an early study, McAdam and Seales (1969) manipulated the level of motivation of subjects performing simple spontaneously triggered movements through monetary reward. In the motivated condition, the BP was larger than in the non-motivated condition.

However, one cannot exclude that motivational effects may be confounded with attentional ones. For example, in this experiment, subjects were told that a reward would be delivered for each “correct” button press, signaled by an auditory tone. However, as indicated by the authors “These instructions were kept vague and subjects were allowed to form their own hypotheses about what might be ‘correct’ response…” (McAdam and Seales, 1969, p. 74). As a consequence, an alternative interpretation in terms of attention cannot be excluded: subjects might have allocated more attention to their response in the reward condition than in the standard one (without good or bad response and no reward), in order to guess which movement characteristic might be followed by a “correct” (reward) signal.

Nevertheless, the motivational context did affect the activity of motor areas, as revealed by the BP preceding these simple spontaneously triggered movements, either directly or indirectly through motivation-induced attentional involvement.

### Attention

If subjects producing short sequences of unilateral spontaneously triggered simple movements are required to perform a concurrent task, the BP is reduced if the concurrent task requires a high attentional load (Baker et al., 2011). This decrease cannot be attributed to a general reduction of cortical activity, since decrease was maximal over central areas for the early BP, and specific to central and ipsilateral sites for the late BP; no effect of attentional load was evidenced at contralateral sites. Although it is always risky to draw strong inferences from surface potential data (Nuñez, 1981; Kayser and Tenke, 2015; Vidal et al., 2015), it is likely that these effects were attributable to a decrease in SMA activity, in line with fMRI data indicating an increase of movement-related SMA BOLD response when subject pay attention to their intention to move (Lau et al., 2004).

Going a step further, Rigoni et al. (2013) attempted to precise the dynamics of cortical activation in the task used by Lau et al. (2004): a variant of Libet et al. (1983) paradigm. Subjects simply pressed a key at the time of their own choosing while watching a rotating clock hand. They were instructed either to pay attention to their intention to move by reporting the moment (position of the clock hand) when they had the intention to act (W condition) or to pay attention to their action by reporting the moment when they began moving (M condition). To attenuate spatial and temporal overlapping effects due to volume conduction, the authors resorted to the Laplacian-transformation. Over the SMAs, the BP was much larger in the W than in the M condition and this effect showed up very early. An opposite pattern was observed over M1 contralateral to the response: the late part, and the late part only, of premovement activities was larger in the M than in the W condition.

First, this confirmed that the increase of SMA BOLD activity reported by Lau et al. (2004) in the W condition concerns premovement activity, consistent with the tight focus observed over the SMAs for the early BP in Rigoni et al.’s (2013) data. Moreover, these data evidenced a dissociation between SMA and M1 activities as a function of attentional conditions. Finally, it must be stressed that these differences showed up under precise EMG control with no EMG differences between W and M conditions. Therefore, it was evidenced that the dynamics of motor areas (even M1) strongly depend on the cognitive state of the subjects, although the executed movements and their motor commands were identical.

### Intentionality

Since the first article of Libet et al. (1983), showing that BP onset precedes the conscious decision to move, there has been a long-lasting, yet unresolved, debate regarding the functional significance of this observation. However, whatever the final outcome of this controversy, it has been demonstrated that instructions regarding the decision influence the BP dynamics. Keller and Heckhausen (1990) compared the BPs preceding involuntarily and voluntarily triggered movements. Involuntary movements consisted in irrelevant slight movements of the finger hand or wrist, performed automatically while subjects were involved in a counting task (counting backward from 3521 in steps of 3). Voluntary movements were obtained in a replication of Libet et al. (1983) paradigm. The voluntary movements evoked larger BPs. Now, voluntary movements were accompanied by larger EMG activity; it is well established that there is a monotonic relationship between the size of the EMG burst and the produced force (Bigland and Lippold, 1954; Bigland-Ritchie, 1981). Given that increased movement force is associated with larger BPs (Kutas and Donchin, 1974) this effect might have corresponded to a by-product of movement force. However, this interpretation is unlikely because the effect of intentionality was confined to midline frontocentral electrodes, indicating either that the involved generators were recruited differentially for involuntary and voluntary movements, or even that midfrontal generators were inactive before involuntary movements.

Up to now, elementary cognitive states such as decisional context, attention motivation, or intentionality have been shown to influence the dynamics of the recruitment of motor areas before movements. More complex cognitive states such as personal belief may also have a strong influence. Rigoni et al. (2011) asked two groups of subjects to read two different texts, one of which had been shown to induce disbelief in free will, the other one being neutral to this respect.

**FIGURE 8:**
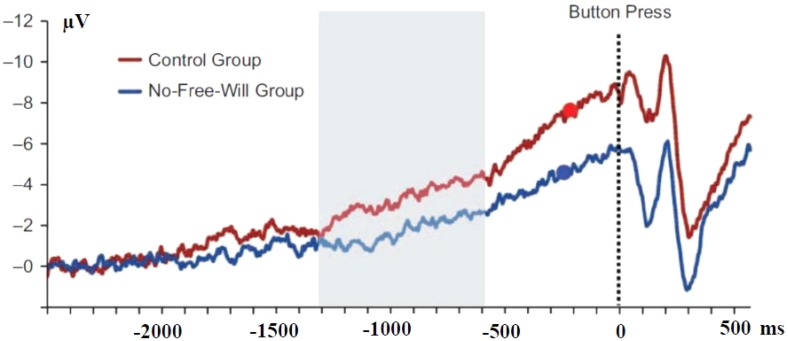
Effect of belief in free-will on the amplitude of the BP. Adapted from Rigoni et al. (2011). Response-locked movement-related activities in control subjects (red line) and subjects who red a text inducing disbelief in free-will (blue line). The red and blue dots indicate the time of decision in control and No-Free-Will groups, respectively. Differences became significant during the period indicate in gray.

Afterward, using the Libet et al. (1983) paradigm, subjects had to produce self-paced simple movements and report the moment when they decided to act. The “disbelief group” showed reduced BP amplitudes as early as one second before the reported time of intention to act (**Figure 8**). Moreover, early (but not late) BP amplitudes (assumed to be generated by medio-frontal structures) correlated negatively with subjects personal free will disbelief scores. Interestingly, disbelief had no influence on the reported time of intention to act.

How can such elaborated cognitive context such as beliefs influence the dynamics of motor structures, remains an open question. One might speculate, for instance, that these effects are achieved via less motivational and/or attentional involvement. In any event, whatever the final answer to this question, it remains that “… beliefs about free will can change brain processes related to a very basic motor level …” (Rigoni et al., 2011, p. 617).

To conclude, it appears clearly that the way we do does not solely depend on what we do. It also depends on our knowledge regarding the circumstances in which we must do. More specifically, motor structures and motor processes are permeable to cognitive operations; motor processes are very sensitive to the influence of cognitive operations and might, as well, contribute to elementary aspects of cognition. Finally, motor structures being a final pathway of several cognitive operations, they can be studied not only for themselves, but also to probe the nature of the upstream cognitive operations that finally recruit them.

## Author Contributions

All the authors participated to the redaction of this article.

## Conflict of Interest Statement

The authors declare that the research was conducted in the absence of any commercial or financial relationships that could be construed as a potential conflict of interest.

## Footnotes

1 Moreover, most often, non-lateralized information regarding the activation of mediofrontal motor structures is lost.
